# The Dysbiosis and Inter-Kingdom Synergy Model in Oropharyngeal Candidiasis, a New Perspective in Pathogenesis

**DOI:** 10.3390/jof5040087

**Published:** 2019-09-21

**Authors:** Martinna Bertolini, Anna Dongari-Bagtzoglou

**Affiliations:** Department of Oral Health and Diagnostic Sciences, School of Dental Medicine, University of Connecticut, Farmington, Connecticut, CT 06030, USA; martinnabertolini@gmail.com

**Keywords:** *Candida*, bacteria, dysbiosis

## Abstract

As more information emerges on oral microbiota using advanced sequencing methodologies, it is imperative to examine how organisms modulate the capacity of each other to colonize or trigger infection. Most mouse models of oral *C. albicans* infection have focused on interactions with single bacterial species. Thus, little is known about the microbiome-mediated interactions that control the switch of *C. albicans* from commensalism to infection. Evidence is accumulating that in immunosuppression where mucosal candidiasis is more prevalent, there is an altered oral bacterial microbiome with reduced diversity, but not an altered mycobiome. Oropharyngeal candidiasis in immunosuppressed humans and mice is associated with a further reduction in oral bacterial diversity and a dysbiotic shift with significant enrichment of streptococcal and enterococcal species. Our recent studies in a cancer chemotherapy mouse model supported the combined profound effect of immunosuppression and *C. albicans* in reducing oral bacterial diversity and provided the first direct evidence that these changes contribute to pathogenesis, representing dysbiosis. There is still a gap in understanding the relationship between *Candida* and the oral bacterial microbiome. We propose that certain oral commensal bacteria contribute to fungal pathogenesis and we identify gaps in our understanding of the mechanisms involved in this cooperative virulence.

## 1. Introduction

Oropharyngeal candidiasis is the most prevalent fungal infection in patients with weakened or immature immune systems, such as HIV+ children, neonates, and patients with malignancies [1,2,3]. Persistent oropharyngeal and esophageal thrush is refractory to most antifungals and a significant clinical problem [4,5]. In states of severe immunosuppression these infections are associated with high morbidity and may lead to systemic disease with mortality ranging from 25–30% [6]. In patients treated with high dose cancer chemotherapy regimens cytotoxic damage to the oropharyngeal, esophageal, and gastrointestinal mucosae combined with myelosuppression, are thought to promote invasion of the mucosal barriers by *Candida* hyphae and ultimately dissemination via the hepatic portal vein to the liver [7,8]. In most immunosuppressed states the main portal of entry of *C. albicans* into the blood vessels is through the esophageal and gastrointestinal epithelium, while the oropharyngeal mucosa is thought to be a fungal reservoir seeding the lower gastrointestinal tract and alveolar mucosa [9]. Because the majority of such systemic *Candida* infections are acquired across the alimentary tract [9,10], it is of paramount importance to discover new approaches to treat or prevent these infections.

It is widely accepted that in a healthy host, unperturbed resident commensal bacterial communities play an important role in limiting *C. albicans* colonization in mucosal sites. This may be accomplished by direct fungal–bacterial cell interactions involving secreted bacterial products, metabolic interactions, or indirectly by influencing the host response [11]. However, epidemiologic evidence for a significant effect of prolonged treatment with antibiotics on the incidence of oropharyngeal candidiasis is not strong and weakens the notion that homeostasis can only be maintained by unperturbed local bacterial communities. In fact, experimental evidence on the use of antibiotics in murine models shows that although antibiotics significantly increase oral fungal burdens, infection as evidenced by pathologic changes in the oral mucosa, requires some form of immunodeficiency [12]. On the other hand, several recent studies using murine models of oral infection have challenged the traditional thinking that most ubiquitous commensal bacteria have antagonistic relationships with *C. albicans*. Indeed, these studies showed that certain oral streptococci have pathogenic synergy with *C. albicans* leading to more severe oral opportunistic infections [13,14,15,16,17,18]. Most of these studies focused on individual bacterial species and very few investigated the interactions between *C. albicans* and resident mucosal bacteriomes in health and disease. In this perspective we briefly highlight our recent work in a mouse model which focused on interactions of *C. albicans* with resident oral mucosal bacteria and review recent evidence from human studies linking oropharyngeal candidiasis (OPC) to a dysbiotic shift of the oral bacterial microbiota.

### 1.1. Oral Mucosal Homeostasis, Relationships between Resident Bacterial Microbiota and C. albicans in Health

The oral cavity is home to hundreds of bacterial species and close to one hundred fungal species [19,20]. *C. albicans* is the most abundant fungal species in the oral microbiome [20,21,22], colonizes the alimentary tract of 30–70% of healthy individuals within the first few weeks of life, and persists without causing disease [23]. The frequency of colonization, number of species, and strains of oral *Candida spp*. vary with age [24]. Interestingly, aging individuals with higher *Candida* load have a lower bacterial diversity in their salivary microbiome and a distinct bacterial composition dominated by streptococci [25]. This positive relationship between *C. albicans* and streptococcal mucosal colonization is corroborated by multiple studies reporting that in women of reproductive age vaginal carriage of *C. albicans* is an independent risk factor for vaginal colonization by group B *Streptococci* [26,27,28]. 

We conducted the first experimental study that examined the influence of *C. albicans* colonization on mucosal resident bacteria of mice with unperturbed indigenous microbiota [12]. Mice are not naturally colonized with *C. albicans*, although they may harbor other indigenous species [29]. Our studies showed that daily oral inoculation of C57B/L6J mice with different *C. albicans* strains induced a decrease in bacterial diversity in the oral mucosa. Surprisingly daily inoculation with *C. albicans* had an impact on oral biodiversity even though fungal colonization was below the sensitivity limit of the CFU assay in most healthy mice. Lower diversity was associated with an increase in the relative abundance of the genus *Enterococcus* and a decrease in *Lactobacillus*. This positive effect of *C. albicans* inoculation on *Enterococcus* species was evident even with filamentation- defective strains associated with lower colonization. In contrast in the jejunum of the same mice, *C. albicans* colonization caused an increase in bacterial diversity, suggesting that the effects of *Candida* colonization on the local bacterial communities are site-specific [12]. Our findings in the small intestinal mucosa were in agreement with older studies showing that low level colonization of the murine cecum of healthy C57B/L6J mice by *C. albicans* leads to shifts in the community structure such that bacterial communities in colonized mice are distinct from and more diverse than naive mice [30]. 

Prior to our work, the role of indigenous bacteria in alimentary tract colonization was studied using combinations of broad-spectrum antibiotics in healthy mice and monitoring the growth of both bacteria and *Candida* in the post-antibiotic period. Enhanced oropharyngeal and intestinal colonization of *C. albicans* was noted with most broad-spectrum antibiotics in mice [30,31,32,33]. However, none of these studies examined the parallel growth of indigenous bacterial communities in the oropharyngeal region post-antibiotics. These studies revealed that a rise in *Enterococcus* species in the stomach and small and large intestine [30,32,33], and *Streptococcus* species in the colon [32] in the post-antibiotic period were associated with increased *C. albicans* colonization. On the other hand, abundance of *Lactobacilli* was inversely associated with *C. albicans* colonization in the lower GI-tract mucosa [30,32]. Importantly, using innovative predictive statistical models, Shankar and colleagues [32] showed greater dependence of *Candida* colonization in the murine ileum and colon on certain bacterial genera such as streptococci than on the mucosal cytokine response in both sites. It is also well-established that commensal anaerobic bacteria are critical in limiting *Candida* intestinal colonization in mice, and colonization levels are generally proportional to the level of antibiotic depletion of anaerobic bacteria [34]. However, such an antagonistic relationship between oral anaerobic bacteria and *C. albicans* colonization has not been established in the healthy murine oral mucosa.

### 1.2. Immunocompomised Hosts Have Altered Oral Bacterial Microbiomes but Not Significantly Altered Mycobiomes

All aspects of *C. albicans* virulence and pathogenesis must be examined in the context of the specific host immune status, since most mucosal infections with this organism afflict immunocompromised hosts. Three well-recognized human immunodeficient states that are risk factors for candidiasis are chronic use of corticosteroids, intensive cancer chemotherapy, and advanced AIDS [3,7,35,36,37]. In addition to allowing *C. albicans* overgrowth leading to infection, immunosuppression may change the overall microbial equilibrium by allowing certain bacterial species to overgrow as well. We conducted the first comprehensive evaluation of the effect of long- term immunosuppression on the oral bacterial microbiome in solid organ transplant recipients using high throughput sequencing of salivary 16S rRNA gene amplicons [38]. Ninety percent of these patients were on chronic corticosteroid use. Compared to a control group, transplant status significantly influenced salivary bacterial community membership with the corticosteroid dose showing significant correlation with bacterial richness and the relative abundance of several bacteria genera. *Enterococcus faecalis* frequency and abundance was significantly increased in transplant patients, together with *Pseudomonas*, *Acinetobacter*, and *Staphylococcus* species. Network correlation analysis also showed abundance of mitis group *Streptococci* to be positively associated with transplant status and with opportunistic pathogens such as *Enterococcus faecalis* [38]. In a similar study of the colon microbiome in transplant patients, major shifts were also reported with a predominant increase in the proportion of *Enterococci* and a decrease in other Firmicutes, evident as early as the immediate post-transplant period when these patients are extremely susceptible to gastrointestinal candidiasis [39]. Finally, a study conducted in lung transplant recipients showed distinct shifts in the oropharyngeal bacterial communities associated with immunosuppression, with the vast majority of patients being co-colonized with an increased abundance of *Streptococci* and *Candida* species [40].

A recent prospective study in a cancer cohort showed that the salivary bacterial microbiome was significantly altered during chemotherapy with enrichment of several Gram-negative species [41]. Chemotherapy disrupted the oral microbiome profoundly, with salivary bacterial communities showing a decrease in diversity correlating with the dose of the cytotoxic drug 5-fluorouracil (5-FU). Oral microbiome shifts could not be explained by antibiotic intake or by a selective antibacterial action of 5-FU [41]. Interestingly, salivary fungal communities were not disturbed by chemotherapy while antibiotic use was not correlated with the risk for developing oropharyngeal candidiasis [22,41]. A chemotherapy-induced increase in oral commensal bacterial burdens, particularly aciduric bacteria such as *Lactobacilli* and *Streptococci* has also been reported in a chemotherapy treated breast cancer cohort [42]. Moreover, a recent systematic review focusing on evaluation of the impact of chemotherapeutic treatment on the oral microbiota in patients with cancer showed that during chemotherapy, there is an increase in bacteria of the *Enterobacteriaceae* family and in *Streptococcus* species, potentially contributing to local oral manifestations, such as oral mucositis, or even systemic infections such as septicemia [43]. Finally, HIV infection was recently associated with lower richness and diversity estimates in the oral bacterial microbiome, with several taxa having increased abundance in this host background. However, as in the cancer chemotherapy cohort above, in this cohort there were no major oral fungal taxonomic shifts associated with HIV infection [44].

The type of immunosuppression can play a decisive role in modifying the local microbial environment where *C. albicans* infections occur, since different types of immune deficiencies may drive qualitatively different bacterial community shifts. For example, mice given cortisone have an increased number of functionally competent infiltrating neutrophils, whereas mice given cytotoxic chemotherapy are severely neutropenic [12,13,45,46]. It is thus possible that bacteria that are primarily cleared by neutrophils overgrow during cytotoxic chemotherapy treatment, whereas neutrophil-resistant bacteria are more likely to survive during chronic use of corticosteroids. In support of this hypothesis, we recently showed that both cortisone and cytotoxic chemotherapy treatment with 5-FU cause a significant increase in the total bacterial biomass on the mouse oral mucosa [12]. However, the effects of these two immunosuppressive treatments on certain mucosal bacteria were different. Whereas in cortisone-treated mice the oral enterococcal biomass decreased, in 5-FU-treated mice the enterococcal biomass increased compared to untreated control mice [12]. This could be explained by the central role of neutrophils in the control of endogenous enterococcal overgrowth in mice [47]. 

Although the mouse and human oral commensal microbiota share very few similarities in health [48], there is increasing evidence that in states of immunosuppression lower abundance oral commensals such as *Enterococci* are selectively enriched in both mice and humans [12,38,39]. Further systematic metagenomics studies are needed to identify global mucosal bacterial community differences in different mucosal sites and different immunosuppression states and mechanistically link these differences to the antimicrobial effector or regulatory functions of immune or mucosal epithelial cells affected by immunosuppression.

## 2. Oropharyngeal Candidiasis as a Result of a Dysbiotic Oral Microbiome

### 2.1. Synergism between Candida and Oral Opportunistic Bacteria in Mouse Models

Based on the studies above it is evident that oral and other mucosal sites along the alimentary tract provide a shared ecological niche for members of the bacterial genera *Enterococcus*, *Streptococcus*, and *Staphylococcus*, with *C. albicans*. Since immunosuppression can enrich the abundance of these bacteria on mucosal surfaces [38,39,40] as well as increase the risk of oropharyngeal candidiasis, this may have important implications in fungal pathogenesis. 

Mounting experimental evidence in murine immunosuppression models supports a synergistic role of oral *Streptococci* with *C. albicans*. Using a cortisone immunosuppression model, we revealed mutualistic relationships between *C. albicans* and the mitis group member *Streptococcus oralis*. We showed that when *C. albicans* is co-inoculated with *S. oralis*, there is an increase in oral mucosal biofilms and *Candida* virulence as evidenced by increased mucosal fungal invasion [13,15,16,17,49]. Our work further showed that *S. oralis* synergizes with *C. albicans* to augment virulence directly by transcriptional activation of the Efg1 filamentation pathway. In particular, *S. oralis* promoted *efg1*- mediated filamentous growth of *C. albicans* and increased *efg1*-dependent *C. albicans als1* gene and protein expression on the surface of hyphae, enhancing interspecies co-aggregation and streptococcal colonization of the oral mucosa [17]. We also found that in orally co-inoculated cortisone-treated mice expression of *Candida* secreted aspartyl protease genes (*sap* 2, 4, 5, and 6) was increased relative to mono-infection. To examine the requirement of these proteases in mucosal invasion during co-infection, we used a Δ*sap*2456 deficient mutant which has strongly attenuated virulence in oral models. Surprisingly, in both organotypic and mouse streptococcal co-infection models the Δ*sap*2456 mutant partially regained the ability to form biofilms, invade, and disseminate to distant organs [16]. These studies suggest that virulence factors of *C. albicans* that have been well established in other models, may become redundant in the polymicrobial environment of the alimentary tract, as was also shown recently by Noble and colleagues [50]. To investigate *Candida*- independent factors that enhance fungal mucosal invasion we examined host enzymatic pathways that may play a role in mucosal barrier breach. We discovered that *C. albicans* and *S. oralis* decreased epithelial E-cadherin levels and increased mucosal invasion by synergistically increasing μ-calpain, a proteolytic enzyme that targets E-cadherin and occludin from epithelial junctions [16].

There is also mounting evidence for pathogenic synergy between *C. albicans* and *Staphylococcus aureus* in murine models. *C. albicans* and *S. aureus* synergy was shown in a cortisone oral infection model [51]. In a peritonitis model disease progression and microbial loads in mice infected with both *S. aureus* and *C. albicans* were significantly higher compared to those with monomicrobial infections [52]. In our cortisone oropharyngeal model and the peritonitis model developed by the Noverr lab, pathogenic synergy between bacteria and *C. albicans* was shown to be host-response mediated via induction of a significantly higher proinflammatory response [15,52]. In the peritonitis model, pathogenic synergy was primarily eicosanoid-mediated [52], whereas in our oropharyngeal model, an exaggerated TLR-2-dependent chemokine and neutrophil response was involved in pathogenesis [15]. 

### 2.2. A Mouse Chemotherapy Model Provides Proof of Concept for the Role of Dysbiotic Bacterial Communities in Candida Pathogenesis

In immunocompromised hosts indigenous bacterial species that form mutualistic relationships with *C. albicans* may increase in abundance leading to a well-coordinated dysbiosis which amplifies mucosal damage. To support this concept, we conducted experiments using a cytotoxic cancer chemotherapy model, which recapitulates oral mucosal and bone marrow toxicity in cancer patients receiving 5-FU [45]. When *C. albicans* is orally inoculated indigenous oral bacterial burdens rise in parallel with fungal burdens in mice receiving 5-FU [12], making the model ideal for the study of the role of resident oral bacteria in fungal pathogenesis. 5-FU-treated mice orally inoculated with *C. albicans* gradually develop severe oroesophageal and intestinal candidiasis over the course of 8 days [12,52]. This prompted the longitudinal examination of site-specific indigenous bacterial changes associated with infection. *C. albicans* infection led to a significant further increase in the oral mucosal bacterial biomass compared to 5-FU alone, with a strong positive correlation between fungal and bacterial loads in the same samples. This was contrasted by findings in the jejunum where bacterial loads decreased in response to *C. albicans* infection. 

Time-dependent analysis of beta diversity changes in the microbiomes of these mice showed that *C. albicans* infection caused a profound disruption of the tongue and small intestinal community structure after 6 days of chemotherapy. *C. albicans* infection was associated with reduction in mucosal bacterial diversity in both sites, with indigenous *Stenotrophomonas*, *Alphaproteobacteria* and *Enterococcus* species dominating the small intestinal, and *Enterococcus faecalis* representing >90% of the oral mucosal communities. Endogenous *Enterococci* were identified in mixed tongue biofilms with *C. albicans* using genus and species (*E. faecalis*)-specific FISH probes, and their increase in these biofilms was validated by species-specific qPCR. In these mixed biofilms *C. albicans* was noted invading into the submucosal tongue compartment [12].

To test whether these findings are immunosuppression type-specific we performed similar analyses in the cortisone-associated oropharyngeal candidiasis mouse model. Consistent with the 5- FU model, infection with *C. albicans* in cortisone-immunosuppressed mice caused a further increase in total oral bacterial burdens. However, in cortisone-treated mice with candidiasis the mucosal enterococcal biomass, as assessed by qPCR, was similar to healthy control mice. FISH staining of oral biofilms in these mice showed that the majority of endogenous bacteria forming biofilms with *C. albicans* were *Staphylococci* [12,53]. This finding together with evidence from others that cortisone-immunosuppressed mice inoculated with both *C. albicans* and *S. aureus* show increased oral pathology [51], implicates *Staphylococci* as accessory pathogens in cortisone-associated oropharyngeal candidiasis.

To explore a role of endogenous *Enterococci* in fungal mucosal invasion in the 5-FU model, *E. faecalis* isolates from mice with oropharyngeal candidiasis were co-inoculated with *C. albicans* in organotypic mucosal constructs. These experiments showed increased invasion of mixed biofilms into the submucosal compartment. *Enterococcus* isolates synthesized gelatinase E and degraded recombinant E-cadherin increasing the permeability of oral epithelial cells in a transwell in vitro assay. Importantly, depletion of these organisms with antibiotics in vivo attenuated oral mucosal E- cadherin degradation and *C. albicans* invasion without affecting fungal burdens, indicating that bacterial community changes contribute to pathogenesis and represent overt dysbiosis [12].

### 2.3. Evidence for Bacterial Dybiosis in Oropharyngeal Candidiasis in Humans

In healthy patients with denture candidiasis an enrichment of the tongue mucosa with *Enterococci* and *Streptococci* was recently reported [54]. This finding supports the concept that candidiasis occurs in a polymicrobial environment enriched with these bacteria consistent with our mouse tongue infection models [13,15,16,17,49]. We also conducted the first prospective study of oral microbiome changes in chemotherapy-treated cancer patients who develop oropharyngeal candidiasis [22]. In this cohort, development of oropharyngeal candidiasis was not associated with mycobiome structure shifts but was the result of increased *Candida* load, with *C. albicans* being the most abundant species [22]. Similar to our mouse 5-FU model, in this human cohort consisting of nine patients who developed oral candidiasis, infection was associated with an increase in oral bacterial burdens, albeit not statistically significant. Although the identification of distinct dysbiotic shifts during the development of candidiasis was not possible in this small patient cohort, we were able to link a lower baseline bacterial diversity with a significantly increased risk for infection. In addition, subjects with increased risk were more abundantly colonized by aciduric bacteria including certain *Streptococcus* species. Our findings thus suggested that increased abundance of aciduric bacteria may be a risk factor underlying susceptibility to oropharyngeal candidiasis in chemotherapy. It is also worth noting that the effect of certain bacteriome members on infection risk was greater than the effect of the baseline proportions of *C. albicans* in the oral microbiome. Finally, in a recent study oral bacterial dysbiosis was implicated in oropharyngeal candidiasis in humans with hyper-IgE inflammatory syndrome, with *S. oralis* identified as the top abundant bacterial species during infection [55]. 

Although strong positive associations between *C. albicans* and certain oral bacterial species can be shown in human cohorts with candidiasis, causality is almost impossible to prove. Thus, the question whether certain indigenous bacteria influence *C. albicans* infection or whether changes in the bacterial microbiota are a result of *C. albicans* infection is extremely difficult to answer in human studies, even with prospective study designs and longitudinal repeated sampling of the same individuals. This limitation of human studies strengthens the rationale for asking these questions experimentally using mouse models where *C. albicans* can be introduced de novo and the effects on the local bacterial microbiota assessed. In addition, by conducting bacterial add-back experiments in antibiotics-depleted *C. albicans*-infected mice the effects of different bacterial species on the course of fungal infection can be elucidated. 

## 3. Conclusions and Future Directions

**Figure 1:**
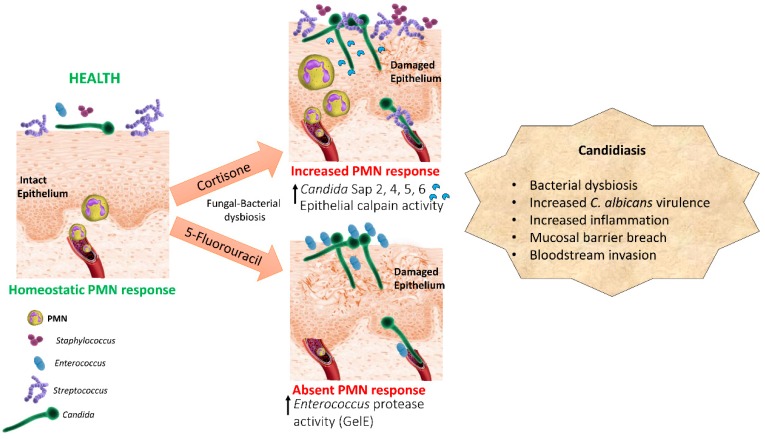
Dysbiosis pathogenesis model of oral mucosal candidiasis in two types of immunosuppression. Cortisone and cytotoxic chemotherapy promote a dysbiotic state characterized by overgrowth of *C. albicans* and different resident or transient oral bacteria, such as *Streptococci, Staphylococci*, and *Enterococci*, which have mutualistic relationships with the fungus. Bacteria act as accessory pathogens by increasing *C. albicans* virulence gene expression, activating host proteolytic pathways or releasing their own proteolytic enzymes which contribute to mucosal barrier breach.

Based on the human and experimental evidence presented above we propose a pathogenesis model in oropharyngeal candidiasis whereby immunosuppression coupled with *C. albicans* overgrowth results in bacterial dysbiosis with dominant species that have the ability to act as synergistic or accessory pathobionts (Figure 1). According to this pathogenesis model immunosuppression may lead to further enrichment with bacterial species which are ubiquitous members of the oral microbiota such as *Streptococci*, or with low abundance transient species not considered part of the healthy oral microbiota, such as *Staphylococci* and *Enterococci* [38]. Given the different effect of cortisone and 5-FU-induced immunosuppression on oral *Enterococci* [12] we propose that the type of immunosuppression influences the type of bacterial dysbiosis associated with mucosal candidiasis. Whether oral bacterial community changes are affected by the type of immunosuppression in humans or mice requires more investigation. More prospective metagenomics studies are needed in humans with elevated risk for oropharyngeal candidiasis assessing both bacterial and fungal genomic components in the same oral samples longitudinally.

Our studies in the cancer chemotherapy murine model underpin the hypothesis that bacterial community changes during infection represent a dysbiotic shift promoting *C. albicans* virulence. In these studies, we identified endogenous *Enterococci* as synergistic pathobionts that augment *C. albicans* mucosal invasion [12]. Like *C. albicans*, *Enterococcus* species are a major concern in patient critical care due to resistance to multiple antibiotics [56]. In the oral cavity of healthy humans *Enterococci* are considered transient commensals and carriage rates are low [57]. However, the oral carriage rate of *Enterococcus* species (predominantly *E. faecalis*) in chemotherapy patients or HIV positive patients rises to 82% [58,59,60]. In particular, in chemotherapy patients both *Candida* and *E. faecalis* abundance increase over time and may place individuals at higher risk for mucosal pathology [61]. These are also some of the most high-risk populations for oropharyngeal candidiasis. Thus, our findings of a mutualistic relationship between these organisms in the murine chemotherapy model are relevant to the human condition and may have serious clinical implications for cancer chemotherapy patients [62,63]. However, more studies are needed to mechanistically dissect the synergistic interactions of *Enterococci* and *C. albicans* in this host background. An older study using live *Enterococcus* organisms in a mouse intraperitoneal infection model showed that *C. albicans* promoted microbial tissue burdens and worsened infection outcomes [64]. There are currently no infection models studying the interaction of these organisms in the oral mucosa and such models are urgently needed.

As shown in our murine models, *C. albicans* breach of mucosal barriers may be exacerbated by mucosal bacteria, in a well-coordinated dysbiosis. There is currently limited information on how interactions of *C. albicans* with the resident bacterial microbiota can affect the oral mucosal barrier. Recent combined genomics and culture approaches have identified 76 culturable bacterial species in the murine lower GI tract [65]. A similar large-scale cultivation study in the murine oral cavity, which would allow more precise taxonomic and functional classification of bacterial sequences is not yet available. Without a comprehensive genomic database of sequences corresponding to murine culturable and uncultured bacterial species the information we glean from murine oral metagenomics studies will be of limited value.

The limited efficacy and increased toxicity of available antifungal drugs, in addition to the emergence of drug-resistance in *Candida* species [66], bring urgency to exploring alternative therapy or preventative strategies against fungal infections. US and international guidelines for the management of oropharyngeal candidiasis in high risk patients include local antifungal treatments as first line treatment, such as nystatin or amphotericin B mouthwashes, or miconazole mucoadhesive tablets. Second line antifungal treatment is usually with an oral systemic azole such as fluconazole [67]. We propose that in addition to addressing the fungal pathogen with such treatments, correcting the underlying bacterial dysbiosis will restrict fungal breach of mucosal barriers leading to bloodstream infections in hosts with weakened immune systems. Animal [68,69] and human studies [70,71] have shown that the daily consumption of probiotic *Lactobacilli* may reduce the oral colonization of *C. albicans*. In vitro studies have shown the ability of different *Lactobacillus* species to have variable effects on the macrophage cytokine response and pattern recognition receptor expression in response to *C. albicans* [72]. Clinical trials are needed with probiotic *Lactobacillus or S. salivarius* strains with a confirmed protective effect in preclinical murine immunosuppression models. Pathogenic synergy between *C. albicans* and oral dysbiotic bacteria also raises the possibility of exploring a convergent immunity approach to develop novel vaccine strategies, as has been reported for *C. albicans* and *S. aureus* [73]. Targeting an epitope from a bacterial species that occupies the same mucosal niche and interacts with *C. albicans* to promote pathogenesis may contribute to overall protective immunity against *C. albicans*.

In conclusion, it is both timely and imperative to consider the pathogenesis of mucosal candidiasis in the context of the physiology of the resident bacterial communities within which *C. albicans* causes disease. In sufficient numbers and in a specific host environment commensal bacteria can become pathobionts and directly or indirectly modulate the virulence of *C. albicans*. Going forward, mucosal infection models should take advantage of the knowledge gained from the metagenomics field to identify resident commensals with the potential to become pathobionts and define mechanisms of pathogenic synergy with *C. albicans* in different host immunosuppression backgrounds. Such studies will be instrumental in devising better preventative and therapeutic strategies in mucosal candidiasis.
